# *Colpodella* spp.–like Parasite Infection in Woman, China

**DOI:** 10.3201/eid1801.110716

**Published:** 2012-01

**Authors:** Cong L. Yuan, Patrick J. Keeling, Peter J. Krause, Ales Horak, Stephen Bent, Lindsay Rollend, Xiu G. Hua

**Affiliations:** Shanghai Jiaotong University, Shanghai, People’s Republic of China (C.L. Yuan, X.G. Hua);; University of British Columbia, Vancouver, British Columbia, Canada (P.J. Keeling, A. Horak);; Yale School of Medicine, New Haven, Connecticut, USA (P.J. Krause, S. Bent, L. Rollend)

**Keywords:** Colpodella, Chromera, Babesia, erythrocyte, Apicomplexa, China, parasites

## Abstract

The phylum Apicomplexa comprises intracellular protozoa that include many human pathogens. Their nearest relatives are chromerids and colpodellids. We report a case of a *Babesia* spp.–like relapsing infection caused by a newly described microorganism related to the Apicomplexa. This case is highly suggestive of a previously undescribed type of colpodellid that infects vertebrates.

The phylum Apicomplexa comprises intracellular protozoa that include human pathogens that cause diseases such as babesiosis, malaria, and toxoplasmosis (*1*). Apicomplexa evolved from algal ancestors, and their nearest relatives are algae (chromerids) and predatory flagellates (colpodellids) (*2**,**3*). We report a case of relapsing infection that has many characteristics in common with babesiosis. Amplification of DNA in blood and molecular phylogenetic characterization revealed a novel nucleotide sequence closely related to *Colpodella* and *Chromera* spp.

## The Case

**Figure 1:**
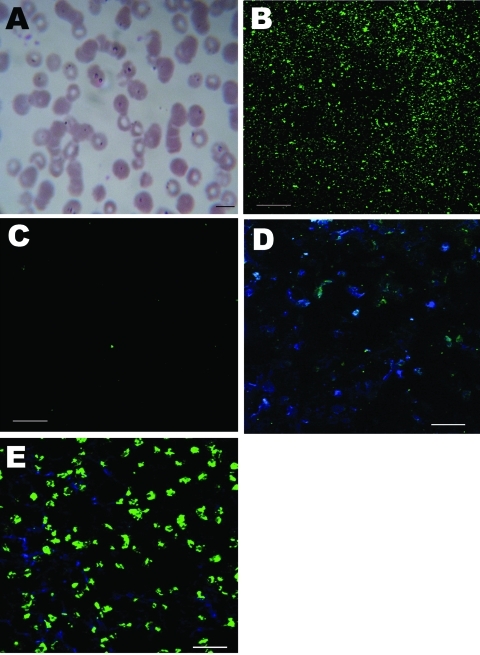
Morphologic appearance of infected erythrocytes of a 57-year-old woman in China and immunofluorescent antibody test results. A) Giemsa-stained thin blood smear showing erythrocyte infected with multiple ring forms (arrowhead). Scale bar = 10 µm; original magnification ×100. B) Patient serum reactive against *Colpodella* antigen. Scale bar = 20 µm; original magnification ×40. C) Healthy control serum not reactive against *Colpodella* antigen. Scale bar = 20 µm; original magnification ×40. D) Patient serum not reactive (green fluorescence) against *Babesia microti* antigen. Scale bar = 20 µm; original magnification ×40. E) *B. microt*i–infected mouse serum reactive against *B. microti* antigen. Scale bar = 20 µm; original magnification ×40.

On March 20, 2008, a 57-year-old woman in the People’s Republic of China sought care for a productive cough and malaise that she had had for 6 months; she was admitted to General Hospital in Kunming City, Yunnan Province. She had evidence of hemolytic anemia with decreased hematocrit and hemoglobin, an elevated reticulocyte count, and elevated lactate dehydrogenase levels. Evaluation of her immune status detected a low percentage of natural killer cells. A peripheral blood smear showed many erythrocytes that contained parasites (Figure 1, panel A). On the basis of its microscopic appearance, the infectious agent was thought to be an *Eperythrozoon* spp. organism, and the patient was treated with intravenous artemether and oral tetracycline for 17 days (*4*).

She had a poor response to therapy and had 4 more episodes of relapsing illness despite a variety of empirically prescribed antimalarial therapies. Parasitemia decreased with each course of therapy but then increased a few days after antimicrobial therapy was stopped.

Blood samples were sent to the Laboratory of Zoonosis and Comparative Medicine, Shanghai Jiaotong University, where *Babesia* spp.–like parasites were identified microscopically. Therapy with atovaquone and azithromycin was initiated, and within a week the parasitemia decreased. The patient subsequently received atovaquone and azithromycin for 8 weeks, and the parasitemia and symptoms resolved completely. She remains asymptomatic 1 year after discontinuation of antimicrobial therapy.

Suspicious *Eperythrozoon* spp. and other bacterial infections were excluded by using a universal primer targeting the 16S rRNA gene (*4*). All published primers for amplification of the specific 18S rRNA region of *Babesia* spp. failed to produce amplification (*5*). To test a wider range of possible candidate species, we aligned all publicly available 18S rRNA sequences from *Babesia* spp. and designed primers to target a highly conserved fragment of the 18S rRNA gene (forward 5′-CCATGCATGTCTMAGTRTAAAC-3′ and reverse 5′-TTCCTCTAAYTGWTAAGGTTC-3′). With these primers, PCR product yielded a 1,653-bp fragment that was cloned and sequenced (triple repeats).

Unexpectedly, the sequence did not closely match any characterized *Babesia* species. Instead, the closest match in BLAST (www.ncbi.nlm.nih.gov/blast/Blast.cgi) analyses based on the entire sequence was *Colpodella tetrahymenae* (89% identity), a member of a genus that is closely related to Apicomplexa but that has never, to our knowledge, been found to infect animals or people (GenBank accession no. GQ411073).

**Figure 2:**
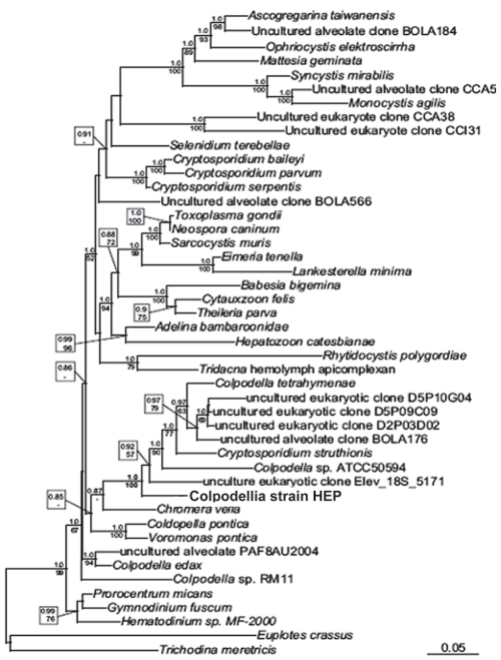
Maximum-likelihood small subunit rDNA phylogenetic tree showing the position of a novel human erythrocytic parasite (in box). Numbers above nodes represent Bayesian posterior probabilities computed by using MrBayes 3.1.2 (www.phylogeny.fr/version2_cgi/one_task.cgi?task_type=mrbayes), with priors set to defaults and a Markov chain Monte Carlo run for 1,000,000 generations of which the first 100,000 were omitted from topology reconstruction. Numbers below nodes depict maximum-likelihood bootstrap support computed from 1,000 replications with the software and model described above. These numbers are bootstrap numbers and refer to the statistical confidence for that node as estimated by the different phylogenetic reconstruction methods. Scale bar indicates 1 base substitution/10 nt.

Phylogenetic trees based on the 18S rRNA were inferred by using Bayesian and maximum-likelihood methods (*6**,**7*). Phylogenetic analyses that included a broad range of eukaryotes confirmed the organism’s overall relationship to the apicomplexan lineage (data not shown). Further analyses included a greater diversity of apicomplexan sequences and showed that it branched at the base of Apicomplexa in the phylogenetic tree. More specifically, in all analyses it appeared to be the sister of a well-supported group consisting of members of the genera *Colpodella* and *Chromera* (Figure 2). On the basis of this position, we refer to the new parasite as colpodellid strain HEP (human erythrocyte parasite).

Convalescent-phase serum from the patient 3 months after the onset of infection was strongly reactive against *Colpodella* antigen; serum from a healthy control was nonreactive (Figure 1, panels B and C). Serum from the patient did not react against *Babesia microti* antigen (Figure 1, panels D and E). These results suggest that the patient’s serum contained antibodies against a *Colpodella* species.

Although the exact diagnosis was not clear until amplified DNA of the intraerythrocytic organism had been genetically sequenced, the patient showed many typical signs and symptoms of babesiosis (*8**–**10*). The relapsing course of illness despite use of antimicrobial drugs that generally are effective against *Babesia* spp. is similar to the course of *B. microti* infection in highly immunocompromised hosts (*8**,**10*). Such patients often require prolonged (at least 6 weeks) antimicrobial therapy to clear infection (*8*).

Phylogenetic analysis of the molecular sequence data show that the patient’s blood contained an organism distantly related to *Babesia* spp. and more closely related to *Colpodella* and *Chromera* spp. Although we cannot rule out the possibility that the intraerythrocytic organisms represent artifact and that the amplified DNA may have resulted from an environmental contaminant, other findings were consistent with the microscopy and PCR findings, i.e., clinical course, response to antiparasitic therapy, and demonstration of antibody against *Colpodella* antigen in the patient’s serum.

## Conclusions

To our knowledge, *Colpodella* spp. have not been shown to infect humans, and it is not surprising that this infection might emerge in an immunocompromised host. Although the patient reported here had no known immunodeficiency syndrome, she was >50 years of age and deficient in natural killer cells. Solitary natural killer cell deficiency is uncommon, and the clinical outcome ranges from no apparent immune deficiency to severe, recurrent, and fatal viral infections (*11*). The effect of natural killer cell deficiency on other pathogens is less clear, although they have been shown to protect against murine malaria (*12*). Colpodellids are microbial predators that feed on various algae and protozoa (*13*). Although they are not known to be parasites, finding a parasitic colpodellid is not entirely suprising. Their mode of predation by use of a primitive apicomplexan infective apparatus is similar to the mechanism by which Apicomplexa enter cells. Thus, the erythrocyte infection by colpodellid strain HEP implies that the feeding apparatus is adept at invading cells as well as extracting their contents.

According to available data, colpodellid strain HEP is best considered to be a new type of colpodellid, but little else is known about it. The manner in which this patient became infected is unknown. During this study, we were alerted to the existence of another new variety of colpodellid that had been isolated from fecal samples of calves with diarrhea in Turkey (A. Cilouglu, pers. comm.). The sequence of the organism isolated from these calves (GenBank accession no. JN245625) is the closest known relative to colpodellid strain HEP (data not shown).

In summary, we describe a case of apparent erythrocyte infection in a human, and the organism’s sequence showed it to be related to colpodellids. Although our findings do not provide conclusive evidence that colpodellids can cause human disease (because the sequence and the infectious agent were not definitively linked and cannot be linked now that the infection has been cleared), no other obvious source for this sequence is apparent.

Colpodellids are not likely to be contaminants in blood samples because they are not common in nature, not known to be associated with humans, and actually rather difficult to maintain in the laboratory. Together with the recent finding of a closely related colpodellid sequence from calves with diarrhea, this case is highly suggestive of a previously undescribed type of colpodellid that infects vertebrates. New studies are needed to further describe this organism and confirm these findings in humans.

## References

[1] Gilles HM, ed. Protozoal diseases. London: Arnold Publishing; 1999.

[2] McFadden GI, Reith M, Munholland J, Lang-Unnasch N. Plastid in human parasites. Nature. 1996;381:482. 10.1038/381482a0 8632819

[3] Moore RB, Obornik M, Janouskovec J, Chrudimský T, Vancová M, Green DH, A photosynthetic alveolate closely related to apicomplexan parasites. Nature. 2008;451:959–63. 10.1038/nature06635 18288187

[4] Yuan CL, Yang ZB, Yao CB, Yang ZB, Zhu JG, Cui L, Prevalence of *Mycoplasma suis* (*Eperythrozoon suis*) infection in swine and swine-farm workers in Shanghai, China. Am J Vet Res. 2009;70:890–4. 10.2460/ajvr.70.7.890 19566474

[5] Kim JY, Cho SH, Joo HN, Tsuji M, Cho SR, Park IJ, First case of human babesiosis in Korea: detection and characterization of a novel type of *Babesia* sp. (KO1) similar to ovine babesia. J Clin Microbiol. 2007;45:2084–7. 10.1128/JCM.01334-06 17392446 PMC1933034

[6] Stamatakis A. RAxML-VI-HPC: maximum likelihood–based phylogenetic analyses with thousands of taxa and mixed models. Bioinformatics. 2006;22:2688–90. 10.1093/bioinformatics/btl446 16928733

[7] Huelsenbeck JP, Ronquist F. MRBAYES: Bayesian inference of phylogenetic trees. Bioinformatics. 2001;17:754–5. 10.1093/bioinformatics/17.8.754 11524383

[8] Krause PJ, Gewurz BE, Hill D, Marty FM, Vannier E, Foppa IM, Persistent and relapsing babesiosis in immunocompromised patients. Clin Infect Dis. 2008;46:370–6. 10.1086/525852 18181735

[9] Zintl A, Mulcahy G, Skerrett HE, Taylor SM, Gray JS. *Babesia divergens*, a bovine blood parasite of veterinary and zoonotic importance. Clin Microbiol Rev. 2003;16:622–36. 10.1128/CMR.16.4.622-636.2003 14557289 PMC207107

[10] Krause PJ, Lepore T, Sikand VK, Gadbaw J Jr, Burke G, Telford SR III, Atovaquone and azithromycin for the treatment of human babesiosis. N Engl J Med. 2000;343:1454–8. 10.1056/NEJM200011163432004 11078770

[11] Fischer A. Human primary immunodeficiency diseases. Immunity. 2007;27:835–45. 10.1016/j.immuni.2007.11.012 18093537

[12] Ing R, Stevenson MM. Dendritic cell and NK cell reciprocal cross talk promotes gamma interferon-dependent immunity to blood-stage *Plasmodium chabaudi* As infection in mice. Infect Immun. 2009;77:770–82. 10.1128/IAI.00994-08 19015248 PMC2632015

[13] Leander BS, Keeling PJ. Morphostasis in alveolate evolution. Trends Ecol Evol. 2003;18:395–402. 10.1016/S0169-5347(03)00152-6

